# Introducing the novel Cytoscape app TimeNexus to analyze time-series data using temporal MultiLayer Networks (tMLNs)

**DOI:** 10.1038/s41598-021-93128-5

**Published:** 2021-07-01

**Authors:** Michaël Pierrelée, Ana Reynders, Fabrice Lopez, Aziz Moqrich, Laurent Tichit, Bianca H. Habermann

**Affiliations:** 1 Aix-Marseille University, CNRS, IBDM UMR 7288, Computational Biology Team, Turing Centre for Living Systems (CENTURI), Marseille, France; 2 Aix-Marseille University, CNRS, IBDM UMR 7288, Team Chronic Pain: Molecular and Cellular Mechanisms, Turing Centre for Living systems (CENTURI), Marseille, France; 3 Aix-Marseille University, INSERM, TAGC U 1090, Marseille, France; 4 Aix-Marseille University, CNRS, I2M UMR 7373, Turing Centre for Living Systems (CENTURI), Marseille, France; 5 Aix-Marseille University, CNRS, IBDM UMR 7288, Turing Center for Living Systems (CENTURI), Parc Scientifique de Luminy, Case 907, 163, Avenue de Luminy, 13009 Marseille, France

**Keywords:** Dynamical systems, Time series, Cellular signalling networks, Computational platforms and environments, Data integration, Data mining, Software

## Abstract

Integrating -omics data with biological networks such as protein–protein interaction networks is a popular and useful approach to interpret expression changes of genes in changing conditions, and to identify relevant cellular pathways, active subnetworks or network communities. Yet, most -omics data integration tools are restricted to static networks and therefore cannot easily be used for analyzing time-series data. Determining regulations or exploring the network structure over time requires time-dependent networks which incorporate time as one component in their structure. Here, we present a method to project time-series data on sequential layers of a multilayer network, thus creating a *temporal multilayer network* (tMLN). We implemented this method as a Cytoscape app we named TimeNexus. TimeNexus allows to easily create, manage and visualize temporal multilayer networks starting from a combination of node and edge tables carrying the information on the temporal network structure. To allow further analysis of the tMLN, TimeNexus creates and passes on regular Cytoscape networks in form of static versions of the tMLN in three different ways: (i) over the entire set of layers, (ii) over two consecutive layers at a time, (iii) or on one single layer at a time. We combined TimeNexus with the Cytoscape apps PathLinker and AnatApp/ANAT to extract active subnetworks from tMLNs. To test the usability of our app, we applied TimeNexus together with PathLinker or ANAT on temporal expression data of the yeast cell cycle and were able to identify active subnetworks relevant for different cell cycle phases. We furthermore used TimeNexus on our own temporal expression data from a mouse pain assay inducing hindpaw inflammation and detected active subnetworks relevant for an inflammatory response to injury, including immune response, cell stress response and regulation of apoptosis. TimeNexus is freely available from the Cytoscape app store at https://apps.cytoscape.org/apps/TimeNexus.

## Introduction

Time-series gene or protein expression data can give invaluable insight into the temporal dynamics of biological processes. It informs about the changes in activity of molecular pathways and key players upon a cellular stimulus or helps characterize molecular activity in cyclic processes, such as the cell cycle or the circadian rhythm. Methods and protocols exist to analyze time-series expression data and extract the dynamically expressed genes from a temporal dataset, some of which have been reviewed and compared in^1^. Results from such tools however do not provide insights into the activity of key molecules or pathways at a given time point. Clustering temporal expression profiles of genes is another possibility to analyze time-series data^2^, which is especially useful to follow the trajectory of expression dynamics of genes over time and to identify co-regulated gene groups^3–5^.

Integrating temporal expression data with protein interaction data is more challenging. Generally, the integration of -omics data with interactomes is very useful to gain deeper insight, like identifying dysregulated pathways or gene communities of interest^6–9^. Popular approaches in network analysis combined with expression data include community detection, identification of active subnetworks or of changes in general network features such as centrality measures^10–21^.

However, most approaches in this type of data integration are limited to static interactomes even though the necessity of dynamic interactomes was recognized some time ago^22^. A dynamic interactome can be modeled as a temporal network. In brief, a temporal network can be described as a sequence of static network states ordered in time, whereby each state represents the activity of the network at a given time point. Temporal networks and their usability in different scientific disciplines have been reviewed in^23,24^. In principle, the same network analysis techniques used for static networks can be applied to temporal networks, for instance extracting active subnetworks or detecting communities, identifying important nodes by centrality measures, etc.^25–27^. Yet, by introducing time as a dimension to networks, some measures and concepts from static networks need to be revisited^23^: for example, in a static network, a path exists from A to C via B, if edges connect (A, B) and (B, C). In a temporal network, with changing edge activities, a path from A to C only exists if the edges (A, B) and (B, C) are active in the correct order (edge (A, B) must be active before edge (B, C)). A number of methods for analyzing static networks, such as path-based centrality measures, are therefore being adapted to temporal networks (reviewed in e.g.^23^).

Some approaches have been introduced that enable users to analyze temporal gene expression data by integrating them with an interactome in a dynamic manner. TimeXnet is a stand-alone JAVA application to identify active subnetworks in interactomes based on time-course expression data^28^. TimeXNet assumes cellular responses to be divided into early, middle and late phases. It takes as an input a weighted interactome together with three gene lists representing the active subset of genes at the three given phases (early, middle, late). It will return the predicted active subnetwork together with the flow between the nodes (active genes) in the early, middle and late phases. The output can be directly visualized in Cytoscape in form of a network. While TimeXNet has shown promising results in mouse innate immune response^29^, it allows only three phases, where each gene belongs to exactly one phase that needs to be defined a priori by the user. Thereby, TimeXNet cannot manage more complex dynamic systems. The Cytoscape app DyNet allows to visualize and analyze dynamic molecular interaction networks^30^. It offers interactive visualization of a temporal network as sets of state graphs, allowing re-arranging of the nodes on each state simultaneously. Moreover, network analysis functions are provided, such as comparing attributes (of nodes or edges) over two or more layers or identification of the most dynamic neighborhood by searching for the most ‘rewired’ nodes in the temporal network. The Cytoscape app DyNetViewer^31^ is able to construct, analyze and visualize active temporal networks. It provides four different algorithms for constructing one static active subnetwork for each time point by retaining only the active nodes from a large protein interaction network at that time point. It provides in addition network analysis functions, mostly focusing on centrality measures and graph clustering algorithms of the temporal network. Furthermore, DyNetViewer enables the user to analyze and visualize the resulting active subnetwork. However, its functions are limited to handling one single layer at a time. Therefore, it does not fully apply the principles of temporal networks.

**Figure 1:**
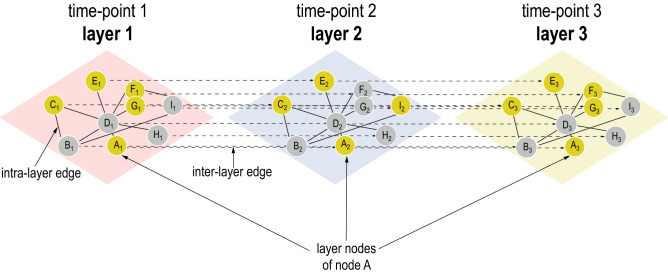
Basic structure of a temporal multilayer network (tMLN). Here shown is a tMLN of three layers. Each layer of the network contains the same protein–protein interaction network (PPIN). Nodes within one layer (*layer-nodes*) are connected via *intra-layer edges*, the same node between two layers is connected by an *inter-layer edge*. For example, the layer-nodes from a given node A (A_1_, A_2_, A_3_) are successively linked by inter-layer edges (A_1_ → A_2_ → A_3_). Numerical data, such as differential expression data from a time-series RNA-sequencing study, are integrated with the TimeNexus tMLN, whereby one layer represents one time point. Yellow nodes represent *query nodes*, which need to be defined a priori by the user. Query nodes can for instance be chosen based on significant differential expression of genes at a given time point versus a control and provided with a node weight. Grey nodes connect query nodes but are themselves not significantly differentially expressed. Intra-layer edges could contain weights in form of confidence values of the given interaction. Inter-layer edges should contain weights describing the change in expression of one node between the two connected layers.

What is generally missing is an easy to use and flexible app for working with temporal data in network analysis. With TimeNexus, we introduce an approach which models a temporal network as a discrete time longitudinal network, in which the expression changes over time are projected on the layers of a multilayer network. Expression changes of one time point are projected on one layer in the form of node weights and the layers are ordered in a time-dependent manner. Other than available methods, TimeNexus uses the edges connecting the layers (*inter-layer edges*) to model transition states between nodes from one time point to the next and thus takes full advantage of the time-series data. A priori, all layers contain the same network (the same nodes and edges) and thus, the multilayer network initially generated by TimeNexus is a multiplex network. TimeNexus multilayer networks are not temporal networks in the sense of^23^, which assumes that edge activity varies over time. To avoid ambiguity, we refer to our networks as *temporal multilayer networks* (tMLNs, Fig. 1). TimeNexus can be used to generate, manage and visualize tMLNs.

We wanted to use TimeNexus to extract active subnetworks from time-series data. Therefore, in the current release of TimeNexus, we provide a connection to the Cytoscape apps PathLinker^16,17^ and AnatApp/the ANAT server^18–21^ for active subnetwork extraction based on differential expression data, making use of their respective programmatic interfaces. PathLinker finds a user-defined number *(K)* of shortest paths between source and target nodes in a network using an improved version of Yen’s k-shortest paths algorithm and then creates active subnetworks by unifying these paths^32^. ANAT, on the other hand, identifies ‘functional networks’ from a large cellular interactome by connecting a set of target proteins (nodes that were for instance identified in a large-scale screen) with ‘anchor’ proteins (nodes around which the network should be constructed). ANAT then minimizes the sum of weights of all edges in the extracted active subnetwork, which is known as the Steiner tree problem^33^. Theoretically, TimeNexus can be extended with any network analysis app available within Cytoscape, provided that it possesses a programmatic interface, as do PathLinker and ANAT.

To test TimeNexus, we used our app together with PathLinker and ANAT on a yeast cell cycle temporal study, following gene expression dynamics of the yeast cell division cycle in synchronized cells^34^. We extracted active subnetworks of the cell cycle from a temporal multilayer network comprised of 16 temporal layers of one full cycle. We scored these active subnetworks for relevance to the process under study by looking for enriched GO-terms related to cell cycle. We also applied TimeNexus to our own data from an injury induced pain assay in mouse, following mechanosensitivity and associated transcriptional changes over 30 days. We predicted pathways relevant for this process, including immune response, stress response, apoptosis regulation and axonal growth. Although TimeNexus has been optimized for temporal and multiplex networks, it is also applicable to all other forms of multilayer networks. TimeNexus is freely available from the Cytoscape App store (https://apps.cytoscape.org/apps/TimeNexus). The source code is also available on GitLab (https://gitlab.com/habermann_lab/temporal-network-project).

## Methods

### Definitions

We project temporal differential gene expression data from a time-series on a multilayer network structure in the Cytoscape app TimeNexus, whereby we assign the differential expression data from each time point to the layer representing this time point in the form of node weights. We refer to this network model as a *temporal multilayer network* (tMLN, Fig. 1). A priori, the network is the same on all layers. Therefore, the tMLN created by TimeNexus is a multiplex network. We refer to a node on an individual layer as a *layer-node*, as opposed to a node of a single-layer static network. Edges connecting nodes within one layer (A_1_, B_1_, C_1_) are termed *intra-layer edges*; those connecting the same node between two different layers (A_1_ and A_2_) are called *inter-layer edges*. Weights can be added to intra- and inter-layer edges. Intra-layer edge weights will most of the time represent confidence scores on a specific interaction and are identical for all layers. Inter-layer edge scores on the other hand can contain information on changes in differential expression of one gene from one time point to the next. Thus, they represent transition weights from one layer to the next and can be used for subsequent network analysis tasks, such as active subnetwork extraction. For follow-up analysis of the tMLN, we furthermore need to define *query nodes*: a query node is a layer-node that shows significant differential expression at the given time point that is associated with that specific layer.

**Figure 2:**
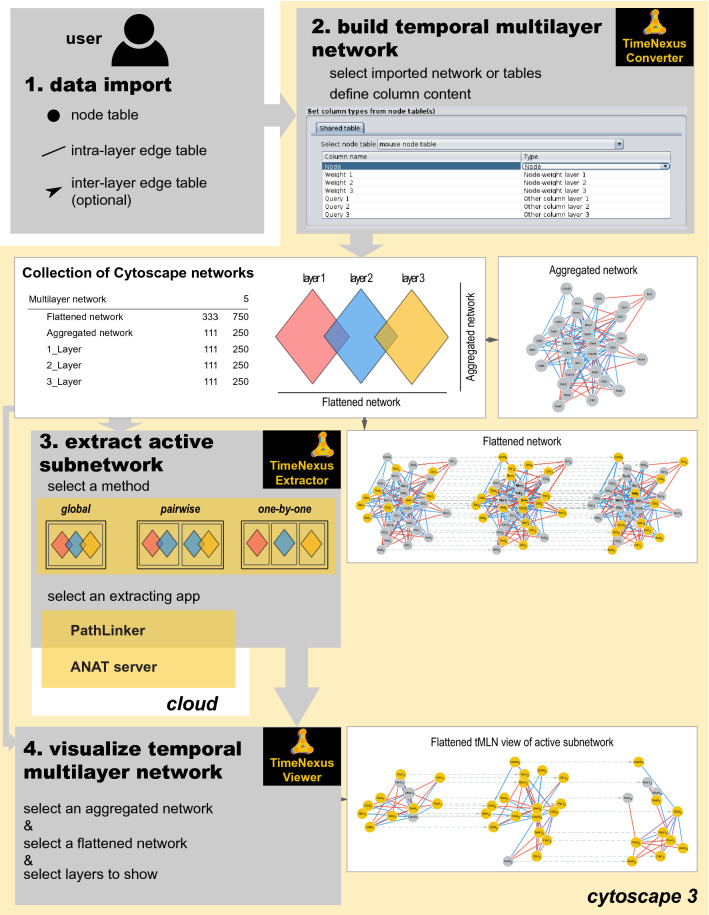
Workflow of the Cytoscape app TimeNexus for creating, managing and analyzing tMLNs. **1. Data import**: First, the elements (layer-nodes, intra-, and inter-layer edges) structuring the temporal multilayer network (tMLN) have to be imported into Cytoscape in the form of tables. **2. Build temporal multilayer network**: In the second step, TimeNexus converts these data into a tMLN. For each element and for each layer, the user selects the appropriate table and specifies the attribute type of each column. Once this is done, TimeNexus represents the tMLN as a collection of Cytoscape networks (center box). It contains a *Flattened network*, an *Aggregated network* and *Layer-specific networks*. In the *Flattened network* view, each layer-node, together with the intra- and inter-layer edges are shown. In the *Aggregated network* view, the layers are collapsed into a single-layer network. **3. Extract active subnetwork**: In the next step, an active subnetwork is extracted from the tMLN. First, the user has to choose the method used to extract active subnetworks. TimeNexus offers three methods: method 1 (*global*): the entire *Flattened network* is used at once, without taking into account the edge type (intra- or inter-layer edges are treated as identical); method 2 (*pairwise*): two successive layers are used to extract the active subnetwork that are then combined to the final active subnetwork; method 3 (*one-by-one*): active subnetworks are extracted in each individual layer and these are combined to the final active subnetwork. For extraction of active subnetworks, TimeNexus offers two algorithms, PathLinker and the ANAT server. PathLinker is a Cytoscape app, while ANAT is executed on the cloud and thus requires a working internet connection. **4. Visualize temporal multilayer network**: Finally, to visualize the tMLN or active subnetwork, TimeNexus creates a view of the *Flattened network*. To do so, it takes the node locations from the *Aggregated network* and transmits it on each layer. Layers are ordered in time on the X-axis from left to right. Visualization can also be done on the full temporal multilayer network. This is however only recommended for smaller networks.

TimeNexus represents the tMLN by two simplified objects: the *Flattened network* and the *Aggregated network* (see Fig. 2 for a representation of an *Aggregated* and a *Flattened network*). These two networks are complementary. Thus, in Cytoscape, a TimeNexus tMLN is represented by a network collection, which includes the *Flattened* and the *Aggregated network*, as well as a static network for each layer, representing the snapshot of differential gene expression at a given time. The *Flattened network* is the visual representation of the tMLN and serves for most applications, such as processing the tMLN by static network tools. In the *Flattened network*, layer-nodes become independent entities and the intra- and inter-layer edges become indistinguishable. Therefore, the Cytoscape ‘create view’ feature will not display this object properly as a temporal succession of layers and a dedicated viewer app is required. The *Aggregated network* represents the collapsed, single-layer network of all layers: all layer-nodes and intra-layer edges are unified in a single node and edge, respectively and all temporal information is lost.

### Temporal information required for building a temporal multilayer network with TimeNexus

TimeNexus builds a tMLN by converting tables into a collection of Cytoscape networks. The conversion requires 2 types of tables: a *node table* containing attributes for each of the layer-nodes and an *intra-layer edge table* connecting the layer-nodes (Fig. 2: **1. data import**). Optionally, an *inter-layer edge table* can be provided which specifies user-defined weight information for connecting the layers.

The node table must contain information on the nodes in form of gene or protein names. It must also contain the information whether a layer-node is a query node or not. The query attribute is important as it is used by the active subnetwork extracting apps to identify the layer-nodes that will contribute to the extracted active subnetwork. The query node attribute can for instance be defined based on the log2 fold change of the layer-node surpassing a selected cut-off and must either be *TRUE* or *FALSE*. Query nodes on each of the layers are thus pre-set by the user. Additional layer-specific attributes such as the weight for each layer-node in form of a numerical value can be provided, for instance reflecting the differential expression at each time point. The interactome of a tMLN is assumed to be the same at each layer. It should however be noted that TimeNexus can also handle multilayer networks that are not multiplex. In this case, the user has to provide one node table for each layer.

The intra-layer edge table contains the information to build the interactome, which is common to each layer. This table contains the edge information of the interacting nodes (proteins or genes). A weight can be given to each intra-layer edge, for instance in form of a confidence score for the interaction. The type of interaction (protein–protein interaction (PPI) or protein-DNA interaction (PDI)) can be distinguished by adding an optional attribute to each edge.

The optional inter-layer edge table has the same format as the intra-layer edge table, but it defines the edges connecting the nodes from one layer to the next. In our example of a tMLN, the inter-layer edges connect the same layer-nodes from two different, consecutive layers. Their attributes represent weights, the so-called inter-layer edge weights, which are calculated by combining the weights of the source and target nodes. Thus, they carry information on the change in expression of that node between two time points. The inter-layer edge weights are used by the extracting apps for identifying active subnetworks. If the inter-layer edge table is not provided, TimeNexus will automatically create these inter-layer edge weights (see below). See Supplementary Tables S1-S3 for examples for the node table and the intra- and inter-layer edge tables. To create a tMLN, at least 2 layers are required.

#### Connecting layers in TimeNexus

The layers are connected through the inter-layer edges. If the user does not provide an inter-layer edge table, the weight between a layer-node on a given layer and its counterpart on the next layer will be computed as

$${\text{w}}_{{{\text{inter - layer}}\,{\text{edge}}}} = {\text{ }}\left( {{\text{w}}\_{\text{i}} + {\text{w}}\_{\text{j}}} \right)/\left( {1 + {\text{w}}\_{\text{i}} + {\text{w}}\_{\text{j}}} \right)$$

where w_i is the weight of the layer-node from the layer i and w_j the layer-node weight on the layer j = i + 1. Contrary to the intra-layer edges, the inter-layer edges are directed for active subnetwork extraction with ANAT. For PathLinker, inter-layer edge directionality is removed, as this app cannot handle mixed edge types.

#### Building, managing and visualizing tMLNs with the Cytoscape app TimeNexus

We created the Cytoscape app TimeNexus to build, manage and visualize tMLNs and to prepare them for extracting active subnetworks (defined as the region of the interactome that connects the differentially expressed nodes over time^12^ (Fig. 2, see also Supplementary Figure S1)). TimeNexus was entirely implemented in Cytoscape 3.8.0^35^ and using Java 11. It is incompatible with earlier versions of Cytoscape.

#### Building the temporal multilayer network (tMLN).

TimeNexus can build a tMLN from scratch by converting tables describing the network structure, or by converting a single-layer network into a tMLN by adding a table with temporal node information. To build a tMLN from scratch, TimeNexus requires at least one node table together with one intra-layer edge table, as well as an optional inter-layer edge table (Fig. 2: **1. data import**). After importing and specifying the content of the tables' columns, the **TimeNexus Converter** that is accessible from the Cytoscape Apps menu creates the tMLN (Fig. 2: **2. build temporal multilayer network**; Supplementary Figure S1) which will appear as a collection of networks within Cytoscape: the *Flattened network*, the *Aggregated network*, as well as one static network for each layer (Fig. 2). The *Flattened network* can be used to visualize the tMLN with the **TimeNexus Viewer.** In this view**,** the layers will be ordered on the X-axis according to time and the layer-nodes will be placed and aligned according to their position in the *Aggregated network.*

#### Extracting active subnetworks from tMLNs using PathLinker or ANAT

TimeNexus can be used to extract active subnetworks. To do so, the methods and apps for extracting the active subnetworks have to be chosen with the **TimeNexus Extractor** (Fig. 2: **3. extract active subnetwork**, Supplementary Figure S1). First, the method for applying active subnetwork extraction on the tMLN needs to be set. There are several possible logical ways to extract active subnetworks from a temporal multilayer network: *globaI*, *pairwise* and *one-by-one* (Supplementary Figure S2). *Global* extracts an active subnetwork from the *Flattened network* representation of the tMLN. In the *global* method, intra-layer and inter-layer edges are not distinguished during the extraction, but are re-established for visualizing the final active subnetwork. This method only considers the queries of the first and the last layer as the source- and target-query nodes, respectively. *Pairwise* combines two adjacent layers in a single network and performs the extraction on this 2-layer *Flattened network*. Each layer is used twice, once as layer N and once as N + 1. The active subnetworks are extracted as in the *global* method for each pair of layers. Finally, all extracted active subnetworks are combined in one final active subnetwork over all time points (layers). *One-by-one* extracts active subnetworks on single layers and combines them at the final step into the final active subnetwork, again over all time points.

Second, the active subnetwork extracting app has to be selected. Currently, the **TimeNexus Extractor** (Fig. 2, Supplementary Figure S1) offers the Cytoscape app PathLinker^17^, which runs in the Cytoscape environment, and the ANAT server^18^, which is called externally, for active subnetwork extraction. PathLinker is called by TimeNexus by its CyRest interface and performs the extraction on the user’s computer. ANAT has a Cytoscape app called AnatApp, but its extraction algorithm is executed on an external server. TimeNexus directly calls this server through a SOAP interface and does not need the AnatApp to be installed to execute ANAT. We only refer to the ANAT server in this paper. When either of the extracting apps is called, TimeNexus displays the specific parameters that need to be set by the user (Supplementary Figure S1). Both apps provide default settings which can be adjusted by the user. For details on the usage and parameter choices of ANAT or PathLinker, the user should refer to the documentation of the respective chosen app. Either all layers of the tMLN or a subset of layers can be selected for active subnetwork extraction. The result of active subnetwork extraction from a tMLN is again a temporal multilayer network. It will appear in Cytoscape as a collection of active subnetworks similar to the network collection described above. It should be noted here that once active subnetworks have been extracted, the tMLN representing the active subnetworks is per definition no longer multiplex, as active subnetworks will have a different number of extracted nodes and edges on each of the layers, depending on the query nodes that have been defined for that specific layer (time point).

#### Visualizing temporal multilayer networks with TimeNexus

Finally, the **TimeNexus Viewer** enables users to visualize a temporal multilayer network. The tMLN can be visualized in several ways (Fig. 2: **4. visualize temporal multilayer network**). In the *Aggregated network* view, all layers are collapsed into a single-layer network. The *Flattened network* shows the individual layers of the tMLN next to each other on a horizontal axis, preserving the position of a layer-node on each layer. The position of a layer-node depends on its position in the *Aggregated network* and layers are connected to each other by the inter-layer edges. Finally, each individual layer can be visualized. We provided a feature to copy the layouts to multiple multilayer networks. It should be noted that the TimeNexus visualization is optimized for networks that have the same semantics, in our case nodes representing proteins or genes and edges interactions between those.

### Yeast and mouse datasets used

#### Yeast cell cycle dataset

The yeast dataset from Kelliher et al.^34^ was retrieved from the NCBI GEO database (GSE80474). We reprocessed the raw fastq files corresponding to the 36 first samples of the wild-type *S. cerevisiae* cultures from 0 to 175 min by mapping the reads to the *S. cerevisiae* S288C genome R64-1-1 with STAR aligner^36^ with default parameters. Raw read counts were determined using featureCounts^37^.

For all following steps, we selected 16 time points representing the first complete cell division cycle. These start at time point 25 min and last until time point 100 min as described by Kelliher according to the expression profiles of key cell cycle regulators. We renumbered these time points in our dataset to start at 0 min of the first full cycle (corresponding to 25 min in the original dataset) until 75 min (corresponding to 100 min in the original dataset). Using edgeR^38^, lowly expressed genes were removed by the automatic function *filterByExpr* and the read counts were normalized by the Trimmed Mean of M-values (TMM normalization), resulting in normalized log-counts per million (logCPM). Then, we calculated the log2FC for each gene at time point i (t_i_) versus its mean over the entire first cycle as follows:

$${\text{log2FC}}_{{{\text{node}}}} \left( {{\text{t}}_{{\text{i}}} } \right) = {\text{logCPM}}_{{{\text{node}}}} \left( {{\text{t}}_{{\text{i}}} } \right){\text{ }} - \left\langle {{\text{logCPM}}_{{{\text{node}}}} } \right\rangle$$

where logCPM is the log-counts per million given by edgeR and < logCPM > is the average logCPM over time for a given gene. Genes with a |log2FC| higher than or equal to 0.25 were considered differentially expressed and defined as query nodes at the respective layer where this cut-off criterion was met. As no replicates were available, we did not consider statistical significance for this dataset.

#### Time-resolved assay and RNA-sequencing dataset of a mouse pain assay

##### Pain assay

All experiments were conducted in line with the European guidelines for care and use of laboratory animals (Council Directive 86/609/EEC). All experimental procedures were approved by an independent animal ethical committee (APAFIS), as required by the French law and conform to the relevant institutional regulations of the French legislation on animal experimentation under the license number 2015070217242262-V5#1537. All experiments were carried out according to the ARRIVE guidelines. C57/Bl6JRj male mice of 8–12 weeks of age were bought from Janvier Labs (https://www.janvier-labs.com). Mice were maintained under standard housing conditions (22 °C, 40% humidity, 12 h light cycles, and free access to food and water). Special effort was made to minimize the number as well as the stress and suffering of mice used in this study.

##### Carrageenan-Induced inflammation

20 µl of a solution containing 1% carrageenan in H2O (weight/vol, Sigma) were injected subcutaneously into the plantar side of the left hindpaw, using a 30G needled syringe. Mechanical thresholds of the plantar surface were determined using Von Frey’s filaments with the up-down method^39^, prior to inflammation (D0) and one- (1d), three- (3d) and thirty-days (30d) post inflammation.

##### RNA extraction

Mice were deeply anesthetized with a mix of ketamine/xylazine and transcardially perfused with 5–10 mL RNA Later (Qiagen). L3 to L5 Dorsal Root Ganglia (DRG) were rapidly dissected and RNA was extracted by using RNeasy Micro Kit (Qiagen), according to manufacturer's instructions. For quality control, RNAs were loaded on an RNA NanoChip (Agilent) and processed with 2100 Bioanalyzer system (Agilent technology).

##### RNA sequencing

DRG RNAs were extracted in experimental duplicates from 2–3 mice each (2 pooled replicates). RNA-seq libraries were prepared using the TruSeq RNA Sample Preparation Kit (Illumina). All libraries were validated for concentration and fragment size using Agilent DNA1000 chips. Sequencing was performed on a HiSeq 2000 (Illumina), base calling performed using RTA (Illumina).

##### Data processing of RNA-seq datasets

Mouse sequencing data were quality controlled using FastQC (https://www.bioinformatics.babraham.ac.uk/projects/fastqc/). We used cutadapt (https://cutadapt.readthedocs.io/^40^,) to trim adapter sequences. Resulting trimmed reads were mapped to the *M. musculus* genome version 10 (mm10) using STAR aligner with default parameters. Mapped data were re-analyzed with MultiQC^41^. Raw read counts were determined and filtered as described above for the yeast dataset. Differential expression analysis was done using edgeR, comparing the time points 1-day post injection (PI), 3 days PI and 30 days PI always against the 0 day control prior to injection. Finally, we showed the evolution of gene expression for the significantly differentially expressed genes of the mouse dataset by first computing the z-score of log counts per million and then splitting the significantly differentially expressed genes according to the time of their significant differential expression. Raw fastq files were submitted to the Gene Expression Omnibus database under the accession number GSE161764. We defined layer-nodes as query nodes if the associated gene had an adjusted p-value lower than 0.05 for that given time point (layer) versus the 0d control. Adjusted p-values were calculated using Benjamini–Hochberg correction^42^.

### Building the *S. cerevisiae* and *M. musculus* interactomes and node tables

Both interactomes were built from the high-quality protein–protein interactions (PPIs) provided by HitPredict^43,44^. As recommended, interactions with a confidence score lower than 0.281 were removed to only keep high-quality interactions. We also removed self-loops in the network. For the yeast interactome, YEASTRACT + protein-DNA interactions (PDIs)^45^ were concatenated with the PPIs to obtain a more complete network. The yeast cell cycle interactome was built by first fetching the 130 genes assigned to the KEGG cell cycle pathway (KEGG pathway sce04111, https://www.genome.jp/pathway/sce04111), and adding the interactions from HitPredict and YEASTRACT + . We used the extracting apps PathLinker and ANAT, both of which do not support multi-edges between nodes. Thus, we merged multi-edges of a given node pair by taking the mean of their confidence scores. For this, we assumed that a PPI is equal to 2 directed edges and set the confidence score of each PDI to 1. The final edge lists gave the undirected intra-layer edge tables. The nodes of the tMLN represent both, the genes and the proteins as the same entities, in case a node is both, a protein in a PPI or a regulated gene in a PDI. Nodes of genes that were not detected in the RNA-seq datasets were removed. Consequently, edges where one partner was removed were also filtered out. The weight for each individual layer-node was computed as follows:

$$w_{{node}} = - {\text{log}}10\left( {{\text{p}}\_{\text{adj}}} \right)*\left| {{\text{log2FC}}} \right|$$

where p_adj is the adjusted p-value and log2FC the log2 fold change for the time point represented by that layer. As no replicates were available for the yeast cell cycle data, the p_adj term was ignored for this dataset. The node weight was then standardized between 0.01 to 1 (a lower bound of 0.01 was chosen to avoid rejection of nodes with weight 0 by extracting apps). Moreover, a layer-node was tagged as a query for a layer if this layer-node had a |log2FC|≥ 0.25 for the yeast cell cycle dataset; or if it had been defined as significantly differentially expressed with an adjusted p-value < 0.05 for the mouse dataset. Node names, node weights, as well as the information whether a node is a query node were contained in the node tables, enabling TimeNexus to set the correct attributes to the layer-nodes.

### Extraction of active subnetworks

Active subnetworks were extracted with TimeNexus in combination with either ANAT or PathLinker from the yeast cell cycle time-series dataset. The algorithm “anchored network” with the sub-algorithm “approximation” was applied for ANAT. The network was set as “undirected” for PathLinker. For performance tests with PathLinker, we selected the optimal parameter K = 750 by testing PathLinker with K-values from K = 50 to K = 2000 and optimizing for the F1-score (Supplementary Table S4; see below for calculating performance measures). PathLinker with a K = 50 was used to extract an active subnetwork for the mouse dataset, as the network size and the number of queries were both smaller. All other parameters were chosen by default.

#### Construction of maximum-weight, node-randomized and weight-randomized networks for robustness tests

To test the robustness of TimeNexus, we generated 3 types of multilayer networks from the yeast cell cycle tMLN. The *maximum-weight* network had intra-layer edge weights of 1 for each connection. The *node-randomized* network had node names shuffled in the node table, so the biological meaning of the network was lost. For the *weight-randomized* network, random weights were assigned to intra-layer edges following the uniform distribution [0.01,1). A lower bound of 0.01 was chosen instead of 0, as ANAT removes edges with weight 0. For all networks, the node and inter-layer edge weights were not changed.

### Calculating performance measures for extracted active subnetworks

We computed the extraction performances by testing if PathLinker and ANAT were able to recover the 130 genes of the KEGG yeast cell cycle pathway sce04111 (https://www.genome.jp/pathway/sce04111)^46^. In each extracted active subnetwork from the yeast cell cycle dataset, we counted the number of nodes in this active subnetwork (# *subnetwork nodes*) and the number of active subnetwork nodes overlapping with the 130 KEGG cell cycle genes (*True Positives (TPs)*). We then calculated the percentage of the active subnetwork size, the False Positives (*FPs*, as *subnetwork size minus TPs*), as well as the false negatives (*FNs,* as *# KEGG cell cycle genes minus TPs*). From these values, we computed a set of scores: the ratio of extracted nodes and the interactome size, as well as Recall, Precision, and F1-score as follows:

$$\begin{array}{*{20}l} {Recall{\text{ }} = {\text{ }}TP{\text{ }}/{\text{ }}\left( {TP{\text{ }} + {\text{ }}FN} \right)} \hfill \\ {Precision{\text{ }} = {\text{ }}TP{\text{ }}/{\text{ }}\left( {TP{\text{ }} + {\text{ }}FP} \right)} \hfill \\ {F1-}{score{\text{ }} = {\text{ }}2{\text{ }}*{\text{ }}\left( {\left( {Recall{\text{ }}*{\text{ }}Precision} \right){\text{ }}/{\text{ }}\left( {Recall{\text{ }} + {\text{ }}Precision} \right)} \right)} \hfill \\ \end{array}$$

In addition, we performed GO enrichment analysis of active subnetworks to test for relevance of extracted nodes for the biological process ‘cell cycle’. The tests were performed using modEnrichr for yeast^47^. We first extracted the expected enriched terms for 130 genes of the KEGG cell cycle pathway. A term was called “enriched” if its adjusted p-value was lower than 0.05. We then computed the percentage of these enriched terms related to KEGG cell cycle genes also found to be enriched for the nodes of the extracted active subnetwork. Finally, we also calculated this percentage of relevant terms at the first quartile (top 25% enriched terms) of the active subnetwork.

### Enrichment analysis of active subnetworks extracted from mouse pain assay data

We used Enrichr^48^ to calculate enrichments for active subnetworks extracted from the tMLN integrating the mouse pain assay temporal RNA-seq data and the mouse interactome. Enriched terms had an FDR < 0.05 and a combined score > 100.

## Results

### Core functions of the TimeNexus app

TimeNexus was developed with the idea to create a versatile framework for working with temporal multilayer networks in the Cytoscape environment (Fig. 2). This included a function to easily create tMLNs given tabular information on the structure of the network and its temporal dynamic – realized in the **TimeNexus Converter**. We wanted to enable users to visualize tMLNs in different ways – realized in the **TimeNexus Viewer**: in form of a *Flattened network*, which visualizes the tMLN itself, as well as an *Aggregated network*, representing the collapsed view of the tMLN. Finally, we wanted to be able to extract active subnetworks from tMLNs. We realized this by connecting TimeNexus to active subnetwork extracting apps available in Cytoscape that have a programmatic interface, PathLinker and the ANAT server. We wanted to take full advantage of the information provided by the temporal multilayer network. We therefore decided to include edge weights for the inter-layer edges of the tMLN that connect the same gene between two layers. These edge weights represent transition weights and describe the change in gene expression of a gene between two consecutive time points. The functionality for active subnetwork extraction was realized in the **TimeNexus extractor**.

We wanted to demonstrate and test the usability of TimeNexus by extracting active subnetworks from two temporal gene expression datasets: 1) yeast cell cycle expression data which offer highly resolved temporal information; and 2) mouse temporal gene expression data following pain response after injury with low temporal resolution.

### Active subnetwork extraction using TimeNexus and PathLinker identifies relevant processes involved in early and late cell cycle events in *S. cerevisiae*

**Figure 3:**
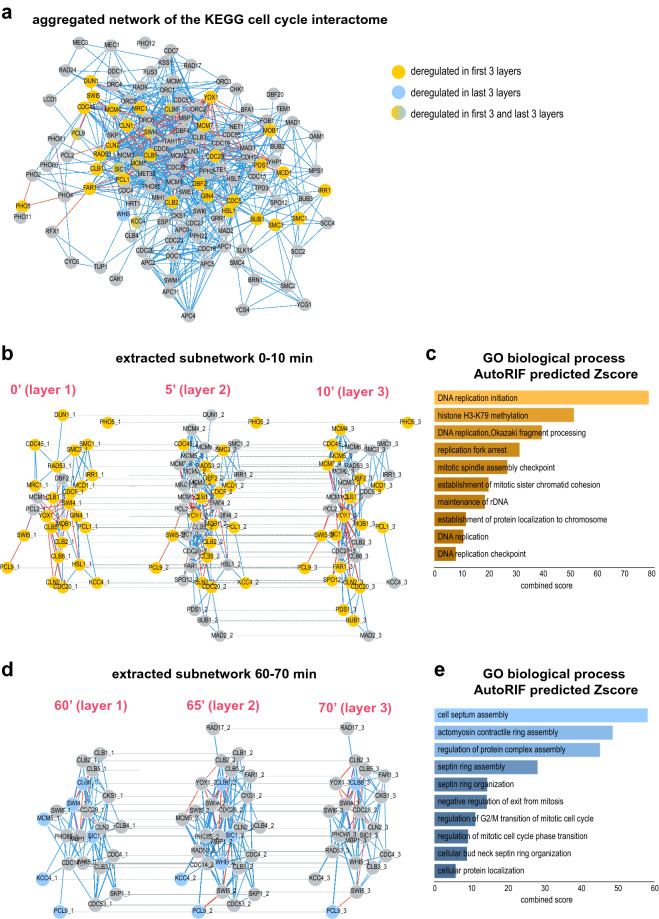
TimeNexus extracts active subnetworks from the yeast cell cycle interactome enriched in relevant biological terms related to cell cycle from early and late cell cycle stages. (**a**) *Aggregated network* of the *S. cerevisiae* cell cycle pathway*,* containing core components of the yeast cell cycle as defined by KEGG. Yellow nodes are differentially expressed query nodes in the first three time points (0 min, 5 min, 10 min) of the first full cycle in the time-series expression dataset^34^, blue ones are differentially expressed query nodes in the late time points 60 min – 70 min; those with a gradient from yellow to blue are differentially regulated and therefore query nodes in both, early and late time points. Blue lines (intra-layer edges) represent protein–protein interactions, red ones protein-DNA interactions. Dotted lines represent inter-layer edges. The interaction data were extracted from HitPredict and the YEASTRACT + databases, respectively. (**b**) An active subnetwork was extracted from the first three time points of the yeast cell cycle (0–10 min), containing genes differentially expressed in early phases of the cell cycle. Layer 1 (0 min): 28 nodes and 58 intra-layer edges; layer 2 (5 min): 41 nodes and 134 intra-layer edges; layer 3 (10 min: 35 nodes ad 95 intra-layer edges. (**c**) Enrichment analysis with genes in the early active subnetwork identified processes related to replication and active transcription. (**d**) An active subnetwork of late time points in the cell cycle (60–70 min) was extracted. Layer 1 (60 min): 22 nodes and 72 intra-layer edges; Layer 2 (65 min): 27 nodes and 101 intra-layer edges; Layer 3 (70 min): 21 nodes and 70 intra-layer edges. (**e**) Enrichment analysis of the genes contained in the late active subnetwork from time points 60–70 min shown in (**d**) resulted in enriched pathways related to late processes in the cell cycle, such as contractile ring organization, cell septum assembly or septin ring assembly and organization. Shown in (**b**) and (**d**) are the extracted active subnetworks of core cell cycle components of the early and late phases as displayed by the **TimeNexus Viewer**. Active subnetworks were extracted using PathLinker (*pairwise* method, K = 150). Enrichment results from the modEnrichR server for yeast were sorted according to the EnrichR combined score^48^. Colorings of enrichment plots were chosen according to the colors of the first 3 (orange) and last 3 (blue) layers.

We wanted to test TimeNexus using a well-described, temporal biological system. We chose the budding yeast cell cycle as our model system. During the cell cycle, cells duplicate their content, replicate their DNA and at the end of the cycle faithfully divide into two identical cells. A cyclin-dependent kinase and its various, successive binding partners, the cyclins, drive progression of the cell cycle by precisely controlled events of phosphorylation, which is followed by the destruction of the kinase activity by the anaphase promoting complex (APC) at the onset of mitosis. Some cell cycle regulators are tightly controlled at transcriptional level. To test TimeNexus, we used time-resolved expression data from a previous study on the transcriptional dynamics of the cell cycle^34^: in that study, *S. cerevisiae* cells had been synchronized before releasing them to undergo three cell divisions. RNA had been extracted each 5 min and subjected to RNA-sequencing to monitor the changes in gene expression during the three cell division cycles. We re-processed the raw read counts and used the normalized counts (see Methods) to calculate the log2 fold change (log2FC) in expression for each gene of a time point versus the mean over one cycle. We created a tMLN of the first full cell cycle, representing time points 25–100 min as described in the original publication^34^. For demonstration purposes, we focused on three early time points of the cell cycle, which are characterized by cell growth and DNA replication (time points 0 min, 5 min and 10 min representing time points 25 min, 30 min and 35 min of the original dataset); and three late time points, which fall into the mitotic phase (60 min, 65 min and 70 min, representing the time points 85 min, 90 min and 95 min of the original dataset; see Supplementary Table S5). We also created a cell cycle interactome by adding HitPredict and YEASTRACT + interactions to the 130 cell cycle genes as defined by KEGG (KEGG pathway sce04111, https://www.genome.jp/pathway/sce04111), resulting in a network of 130 nodes (genes/proteins) and 390 intra-layer edges (interactions, see node table and intra-layer edge table in Supplementary Table S5). We used a |log2FC| cut-off of ≥ 0.25 to define a layer-node as a query node. Using **TimeNexus Viewer**, we created the *Flattened network* of the KEGG cell cycle (Fig. 3 a). We used the *pairwise* method and PathLinker with default settings and a K of 150 to extract an active subnetwork from the three early and late temporal layers, respectively.

The extracted active subnetwork of the early phase of the cell cycle contained 41 nodes and 134 intra-layer edges (Fig. 3b) in the aggregated state. As expected, its members included proteins important for cell proliferation, DNA replication and active transcription, such as the MCM proteins MCM1 – MCM7, the cyclin dependent kinases CLB1, 2, 5, 6 and CLN2, as well as CDC28, CDC45, DBF2, SWI4, SWI5 or SIC1. To systematically identify enriched biological processes or phenotypes, we submitted the proteins of the active subnetwork to modEnrichr for yeast. We found that biological processes and phenotypes associated with early cell cycle phases were predominantly enriched (Fig. 3c, Supplementary Table S5).

To test whether extracted active subnetworks truly reflect cell cycle phases, we also used differentially expressed query genes in the three time points between 60 and 70 min, reflecting the late stages of the yeast cell cycle, where cells prepare to undergo cell division (Supplementary Table S5). The active subnetwork extracted with PathLinker in its aggregated state is substantially different from the one of the first three time points, with only 27 nodes and 101 intra-layer edges (Fig. 3d). In accordance with the late stage in the cell cycle, genes involved in cell septum assembly, bud neck septin ring organization, actomyosin contractile ring assembly, regulation of G2/M transition and other late cell cycle events were enriched (Fig. 3e, Supplementary Table S5). Taken together, TimeNexus provides a versatile and useful platform to construct, manage and visualize tMLNs. By linking TimeNexus to active subnetwork extraction tools such as PathLinker, it is able to extract biologically meaningful, active subnetworks from the tMLN as demonstrated by analyzing time-resolved expression dynamics of the early and late yeast cell cycle.

### TimeNexus performance in identifying relevant active cell cycle subnetworks from the *S. cerevisiae* interactome

We next wanted to test more rigorously the extraction performance of TimeNexus in combination with PathLinker or ANAT on tMLNs. More specifically, we were interested whether we could reliably extract the 130 genes defined by KEGG as being part of the yeast cell cycle pathway (KEGG pathway sce04111, https://www.genome.jp/pathway/sce04111) from the entire yeast interactome using the time-resolved cell-cycle expression data^34^. Log2FC was calculated as described above. The absolute log2FC was used as node weight, to compute inter-layer edge weights and to define layer-nodes as queries when their weight was equal to or higher than 0.25 (Supplementary Tables S6, S7). We built a high-quality interaction network for *S. cerevisiae* which included protein–protein, as well as protein-DNA interactions and contained 5785 nodes and 110 000 intra-layer edges in its aggregated state (see Methods and Supplementary Table S6). We constructed the tMLN using the **TimeNexus Converter** and extracted active subnetworks using PathLinker or ANAT. For PathLinker, the parameter K was set to 750 after optimization (Supplementary Table S4). We calculated the efficiency of both extracting apps by calculating Precision, Recall and F1 score for each time point individually, as well as over all 16 time points. Moreover, we performed GO enrichment analysis with the extracted nodes for each time point, as well as over the entire extracted active subnetwork. We scored the percentage of enriched expected terms, so those identical to the original 226 terms enriched for the 130 KEGG-defined cell cycle genes, as well as the percentage of top expected GO-terms in the first quartile of enriched GO-terms (Table 1 and Supplementary Table S8).

**Table 1:** Efficiency and robustness of TimeNexus-based active subnetwork extraction with PathLinker and the ANAT server over the entire tMLN.

Robustness	Subnetwork size (%)	Recall (%)	Precision (%)	F1-score (%)	Percentage of expected GOs	Percentage of top expected GOs
**TN + PL**						
Original	9.6	45.4	**10.6**	**17.2**	**39**	**71.7**
Maximum edge weight	53.2	**93.1**	3.9	7.6	16.2	34.4
Node-randomized	51.6	83.9	3.7	7.0	15.2	27.3
Weight-randomized	11.9	35.4	6.7	11.3	28	47.6
**TN + ANAT**						
Original	12.3	37.7	6.9	11.7	35.2	**68**
Maximum edge weight	12.4	**41.6**	**7.5**	**12.7**	35.7	**68**
Node-randomized	15.1	41.5	6.2	10.7	29	52.7
Weight-randomized	12.6	40.8	7.3	12.4	**36.4**	67

TN + PL: TimeNexus in combination with PathLinker; TN + ANAT: TimeNexus in combination with the ANAT server.

Bold letters indicate the best performance in the respective comparison and category.

Subnetwork size is calculated as the percentage of nodes retained in the extracted subnetwork from the full interactome, Recall represents the correctly retrieved cell cycle genes, Precision the percentage of cell cycle genes relative to the entire extracted subnetwork and the F1-score the overall accuracy. The percentage of expected GO terms is calculated based on the GO term enrichments of the 130 cell cycle genes (as defined by KEGG) alone, for the percentage of top expected GO terms, we only consider the first quartile thereof.

Generally, we could observe that PathLinker performed better than ANAT with our data. PathLinker extracted an active subnetwork that had 9.6% of the number of nodes of the entire yeast interactome in its aggregated form. The overall Recall of core cell-cycle nodes was 45.4% for PathLinker, though Precision was 10.6% only, leading to an F1-score of 17.2%. 39% of expected GO-terms were found overall, and 71.7% expected GO-terms were retrieved in the first quartile of enriched GO-terms. The active subnetwork extracted by ANAT contained 12.3% of nodes of the total aggregated interactome. ANAT reached a Recall of 37.7%, a Precision of 6.9% and an F1-score of 11.7%. 35.2% expected GO-terms were found overall, and 68% within the first percentile of enriched GO-terms (Table 1). Recall, Precision and F1-score were dependent on the individual time point (layer). They peaked in the earlier phases of the cell cycle and dropped towards the end. This was not unexpected, as the number of query nodes was much lower in late phases of the cycle. Overall, expected GO-terms ranged around 50%, whereby the expected GO-terms in the first quartile seemed to be generally high throughout the entire cycle and with both extracting apps (Supplementary Table S8).

We also wanted to know how sensitive active subnetwork extraction with either PathLinker or ANAT was to changes in the network structure or network attributes. To this end, we first changed the weights of all intra-layer edges to 1 (*maximum edge weight*); second, we shuffled the node names from the node table so the biological meaning of the network was lost, but its topology preserved (*node-randomized*); finally, we used random edge weights following a uniform distribution [0.01,1) for the intra-layer edges (*weight-randomized*). We observed that PathLinker was more sensitive to changes in the network structure or attributes than ANAT (Table 1): ANAT performance was overall in the same range for all extracted active subnetworks, though slightly higher performance could be observed for the maximum edge weight network. PathLinker, on the other hand showed significant differences (Table 1). The maximum edge weight, as well as the node-randomized networks resulted in very large extracted active subnetworks, both containing over 50% of the nodes of the original aggregated interactome. Consequently, Recall was very high (93.1% for maximum edge weight and 83.9% for node-randomized), and Precision very low (3.9 and 3.7%, respectively), resulting in low F1-scores (7.6% and 7.0%, respectively). The weight-randomized network showed general lower performance compared to the original one (Recall 35.4%, Precision 6.7%, F1-score 11.3%), together with a lower percentage of enriched GO-terms relevant to cell cycle genes (28% expected and 47.6% top expected GO-terms). To conclude, TimeNexus in combination with particularly PathLinker was able to extract key cell cycle genes as defined by KEGG as an active subnetwork from the tMLN of the entire yeast interactome based on integrated temporal cell cycle expression data, resulting in a significant enrichment of GO-terms related to cell cycle genes in the active subnetwork.

### TimeNexus combined with PathLinker identifies active pathways relevant for tissue inflammation and repair in time-course expression data of pain induction in mouse

**Figure 4:**
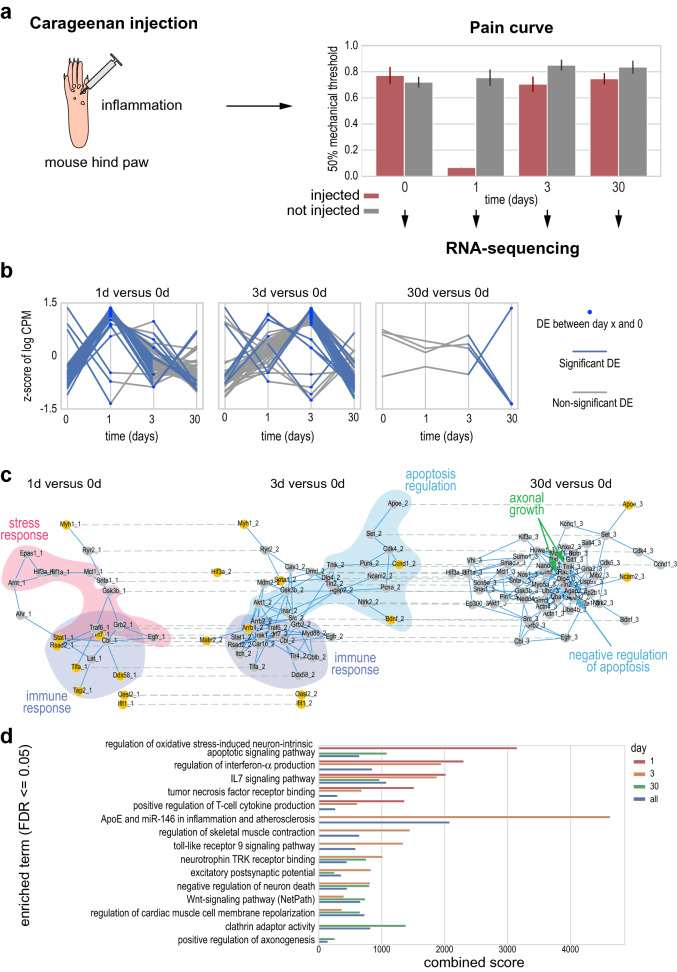
Identification of pathways relevant for cellular stress response, apoptosis, immune response, as well as axonal growth in mouse sensory neurons after Carrageenan-induced inflammation. (**a**) We injected Carrageenan in the hind paw of a C57BL/6 J mouse, which induces inflammation and pain, affecting the sensory neurons. We monitored the mechanosensitivity of the paw before injection, as well as 1, 3 and 30 days after injection. We observed high mechanosensitivity up to day 1. Thereafter, we observed complete recovery of the mechanosensitivity by day 3, which persisted at least until day 30. We isolated the dorsal root ganglions at those time points and performed RNA-sequencing, identifying significant differential gene expression between time points compared to day 0 control (0d, before injection). (**b**) The 3 plots show the significantly differentially expressed genes varying over time. These genes were grouped according to their appearance in the 3 time points, day 1, day 3 or day 30 each compared against the 0d control. Consistent with the onset of injury and inflammation, we could see strong induction of gene expression at day 1, as well as day 3 after injury, while at day 30, only few genes were significantly differentially expressed compared to the 0d control. Genes that are significantly differentially expressed at two time points will be present in each of the two associated plots. Blue dots indicate significant differential expression of a gene at the given time point. Blue lines indicate significant differential expression between two time points. Y-axis is plotted as the z-score of the log-transformed counts per million. (**c**) From a tMLN based on the entire mouse interactome, we extracted an active subnetwork containing 3 layers, one for each time point compared to the 0d control using PathLinker (*pairwise* method, K = 50). We extracted an active subnetwork containing genes relevant for the pain assay: at day 1, we found genes involved in stress (red bubble) and immune response (blue bubble). At day 3, we identified genes involved in immune response (blue bubble), as well as regulation of apoptosis (cyan bubble). Finally, at day 30, a more heterogenous set of genes was identified, including anti-apoptotic genes (cyan arrow), as well as genes involved in axonal growth (green arrows). Orange nodes represent query nodes (which showed significant differential expression at a given time point versus 0d control). Active subnetwork extraction returned Steiner nodes (grey nodes), i.e. nodes that are part of the network, but were themselves not significantly differentially expressed and, thus, not query nodes. Solid blue lines are protein–protein interactions within one layer (intra-layer edges), dashed lines represent inter-layer edges. (**d**) Enrichr enrichment results of WikiPathways and Gene Ontology (GO) Biological Process (BP) and Molecular Function (MF). Nodes from each of the layers (day 1, 3, and 30) as well as the layers of all nodes of the active subnetwork (all) were used for enrichment analysis. Enrichments of the first two time points included terms related to immune and stress response, encompassing signaling pathways involved in these processes. The signature changed at the later time point (day 30), where more terms related to apoptosis, as well as axonogenesis were enriched. Enriched terms had an FDR < 0.05 and a combined score > 100.

Next we tested, whether we could use TimeNexus on other systems, other model organisms and with less dense time-resolved data on differential gene expression. We used our own data from a time-resolved study of recovery from acute pain in mouse. In this experiment, Carrageenan is injected in the mouse hindpaw, inducing inflammation and mechanical hypersensitivity (Fig. 4a). The onset and the recovery from hypersensitivity can be measured by testing the ability of mice to respond to Von Frey filaments with increasing caliber. In this pain model, one day after Carrageenan injection, mice exhibit a significant decrease in their mechanical thresholds, which is a sign of inflammation-induced mechanical hypersensitivity. At day 3 post-inflammation (PI) mice recover normal mechanical sensitivity which remains steady at day 30 PI and beyond (Fig. 4a, Supplementary Table S9). In order to monitor the changes in gene expression in the pain-sensing dorsal root ganglia (DRG), we extracted RNA from these cells and performed RNA-sequencing before (0d), 1 day (1d), 3 days (3d) and 30 days (30d) after Carrageenan injection.

After differential expression analysis, we found that 60 genes were significantly differentially expressed between day 0 and day 1 PI and 38 genes showed significant differential expression between day 0 and day 3 PI (Supplementary Table S9). Finally, only 4 genes were significantly differentially expressed at day 30: Apoe (Apolipoprotein E), Itgb8 (Integrin Beta-8), Ncam2 (Neural Cell Adhesion 2) and Slc25a37 (Mitochondrial Iron Transporter 1). The temporal expression dynamics of significantly differentially expressed genes collected from the 3 comparisons showed that genes were generally upregulated between day 0 and day 1, while the majority of them was downregulated between day 3 and 30. Genes with a significant differential expression between day 30 and day 0 were few (Fig. 4 b). In conclusion, acute pain induced a temporary significant differential expression of genes in DRG and the vast majority of genes returned to basal expression levels after full recovery of the mouse at day 30.

We next were interested whether we could extract active subnetworks relevant for this process from expression data integrated with the mouse PPI interactome using tMLNs. We built a high-quality mouse interactome using HitPredict, containing 3994 nodes and 7296 intra-layer edges in its aggregated form. We created a pain node table from the differential expression data for the three time points 1d, 3d and 30d PI compared to the 0d time point prior to injection. Query nodes were defined as having an adjusted p-value lower than 0.05 (Supplementary Table S9). Using TimeNexus, we generated the tMLN for these data. We used PathLinker with K = 50 and the method *pairwise* to extract active subnetworks (Fig. 4c). Layer ‘1d vs 0d’ contained a network with 23 genes. Among those were genes involved in immune response (Stat1, Irf7, Traf6, Rsad2 and TifA), as well as cell survival and stress response (Arnt, Epas1, Hif3a, Hif1a, Mcl1, Gsk3b, Grb2 and Egfr1, as well as Stat1 and Irf7). At the second time point at 3d versus 0d, genes involved in immune response were still prevalent, as were genes involved in the regulation of apoptosis. The network is less homogenous with respect to pathways at time point 30d versus 0d. We found some genes involved in axonal growth, as well as negative regulation of apoptosis. Finally, we performed enrichment analysis of the entire active subnetwork, as well as the individual time points (1d, 3d, 30d, versus 0d control) and could confirm the enrichment of pathways and GO terms related to immune response and inflammation, regulation of apoptosis, as well as neuronal processes (Fig. 4d, Supplementary Table S9). In order to test, whether active subnetwork extraction from the temporal multilayer network resulted in identification of more, as well as more relevant pathways, we also performed enrichment analysis with all differentially expressed genes identified by differential expression analysis alone (see Supplementary Table S9). We mostly found enriched terms involved in inflammation and immune response at day 1, followed by enrichment of terms related to nerve injury, as well as Wnt and MAPK signaling at day 3. Day 30 with only 4 differentially regulated genes was not further considered in this analysis. The more detailed analysis of the active subnetworks thus helped us identify additional terms relevant for injury and regeneration of DRGs. In conclusion, by extracting active subnetworks from the temporal multilayer network created with TimeNexus, we could identify genes involved in direct response to inflammation, cellular stress and regulation of apoptosis, as well as neuronal processes in DRG following Carrageenan-induced inflammation.

## Discussion

We introduced here TimeNexus, a Cytoscape app to create, manage and visualize temporal multilayer networks. TimeNexus is easy to use: tMLNs can be created either by uploading a collection of tables that contain attributes of the nodes, as well as information on edges; or by adding temporal information to a static Cytoscape network. The **TimeNexus Viewer** allows to visualize the tMLN by creating different views, enabling users to focus on the single static, as well as the dynamic features of the tMLN. This first release of TimeNexus furthermore provides a framework to extract active subnetworks from a tMLN. To this end, we create static networks of the tMLN in three different ways which are standard Cytoscape networks that can be handled by basic Cytoscape features, as well as other Cytoscape apps. These objects are created either *globally* over the entire tMLN by combining all layers in a single-layer network with layer-nodes as separate entities and by ignoring the differences between intra- and inter-layer edges; *pairwise* by creating a single-layer network from two consecutive layers similar to the *global* method, over the entire tMLN structure; or *one-by-one* by creating a single-layer network for each individual layer. The *global* method has the drawback that the network to be analyzed increases drastically, as the initial interactome is multiplied by the number of layers. Active subnetwork extraction is therefore compute-intense. Moreover, only nodes from the first and last layers will be used as source and target nodes and active subnetworks will only be extracted if they span the entire dataset. The *one-by-one* method on the other hand uses less memory, but does not consider inter-layer edges, so the nature of the temporal multilayer network is ignored. The *pairwise* method is a good compromise between both methods and therefore recommended especially with larger networks or many time points. The *global* and *pairwise* method also take full advantage of TimeNexus’ unique feature to work with transition weights between layers, representing expression changes of a gene between two time points. While we have combined TimeNexus with tools to extract active subnetworks from interactomes, it should be noted that any Cytoscape app for network analysis can be combined with TimeNexus, as algorithms are applied to a classical static network structure by the *global*, *pairwise* or *one-by-one* method. The only pre-requisite is the availability of a programmatic interface for the chosen app.

We tested TimeNexus by extracting active subnetworks in combination with the Cytoscape apps PathLinker and the ANAT server. PathLinker outperformed ANAT in extracting biologically relevant, active subnetworks and worked better in our hands. It was however also more sensitive to specific network attributes, such as intra-layer edge weights. This is not surprising, as it uses edge weights to calculate scores for paths between nodes to extract active subnetworks. The user should therefore carefully choose intra-layer edge weights in order to extract meaningful biological information from the network. We also observed that the selection of query nodes has a substantial effect on the results. In general, the overall performance of both extracting apps was mediocre, which might be owed to the test itself: we tried to extract cell cycle genes from the KEGG-defined yeast cell cycle pathway. Many of these genes are not regulated on RNA-level but rather by phosphorylation or protein degradation. While for some processes, RNA- and protein expression levels correlate quite well^49^, this is not necessarily the case for cyclic processes such as the cell cycle, where a rapid activation or destruction of regulatory proteins is required and thus, protein phosphorylation as well as degradation play an important role. However, we did not want to artificially bias the test to extract differentially expressed genes, but rather wanted to know, how efficiently we could recover well-described, core cell cycle genes from the tMLN using either of the two apps, irrespective of their RNA expression dynamics. Therefore, it might not be surprising that both, Recall, as well as Precision were not high with either of the two tested apps. Furthermore, it should be noted that PathLinker and ANAT are optimized to extract active subnetworks from static single-layer networks, not from a temporal multilayer network and thus may not fully consider the information a multilayer network offers.

There are three other Cytoscape apps available for integrating temporal data with interactomes: DyNetViewer, DyNet and TimeXNet. DyNet is not able to extract active subnetworks, which excluded it from further consideration. DyNetViewer creates individual temporal layers from expression data directly, removing all nodes from an interactome that are not significantly differentially expressed. In principle, the output of the DyNetViewer could be used to create directly an active subnetwork within TimeNexus. But this app also omits transition weights from one layer to the next and therefore, is not taking full advantage of the temporal information provided. Yet, its visualization properties exceed those of TimeNexus. TimeXNet can be used for active subnetwork extraction from temporal expression data. However, it defines only three phases, representing early, middle and late genes, which could correspond to the layers in a multilayer network representation. If a higher temporal resolution is required and available, as is the case for a cyclic process such as the cell cycle, the classification in these three phases is difficult to make. Moreover, in TimeXNet, one gene can only be part of one phase, which limits the usability of this tool for cyclic processes even further. We therefore decided not to use it for performance tests, as it would have significant disadvantages compared to TimeNexus in combination with the extracting apps PathLinker or ANAT.

We used TimeNexus in combination with PathLinker to extract active subnetworks from a time-resolved pain assay in mouse, based on expression data from the pain sensing dorsal root ganglia. While we did not find a large amount of significantly differentially expressed genes, we identified by performing tMLN analysis with TimeNexus an active subnetwork that contained genes relevant for the process of inflammation: genes involved in immune response, in cellular stress response and in anti-apoptotic signaling, as well as – at late stages – genes involved in axonal growth. Our active subnetwork contained many Steiner nodes (non-query nodes), representing genes that were not initially identified as significantly differentially expressed. This demonstrates that integrating and analyzing temporal gene expression data together with interaction data leads to meaningful biological insights that can also help in the design of further experimental studies.

We see several possibilities to improve TimeNexus in the future. First, due to the multiplication of the network on temporal layers, the Cytoscape object used for processing and calculation tends to be very large. Thus, a computer with sufficient memory is needed to be able to extract active subnetworks using TimeNexus and the associated apps, PathLinker or ANAT. Programmatically, the TimeNexus object representing the tMLN could be simplified, as the nodes and intra-layer edges are the same on each layer and are just distinguished by their weight. Second, as extracting apps cannot distinguish between intra- and inter-layer edges, it would be desirable to develop a subnetwork extraction algorithm designed to work with tMLNs, which takes full advantage of their inherent temporal information. Finally, TimeNexus in its current form does not have an API, but rather relies on APIs of other Cytoscape apps for its workflow. In future releases, we will provide an API that will allow to include TimeNexus in the workflow of other apps.

In conclusion, TimeNexus is a Cytoscape app that introduces true temporal multilayer networks within the Cytoscape environment. While we have used it to create, manage, visualize and analyze temporal data projected on a multilayer network that is multiplex, it can also handle other kinds of multilayer networks. We have combined the first release of TimeNexus with two apps for active subnetwork extraction, PathLinker and ANAT. However, TimeNexus builds native Cytoscape objects which can be handled by core Cytoscape features or other apps dedicated to network analysis. Therefore, TimeNexus can be extended with other Cytoscape apps, provided they offer a programmatic interface. Consequently, TimeNexus can be added into existing pipelines and workflows as an app for analyzing temporal multilayer networks.

## Supplementary Information

Supplementary Information 1.

Supplementary Information 2.

Supplementary Information 3.

Supplementary Information 4.
