# Supplementary material for: Introducing the novel Cytoscape app TimeNexus to analyze time-series data using temporal MultiLayer Networks (tMLNs)
Source: Sci Rep. 2021 Jul 1;11:13691. doi: 10.1038/s41598-021-93128-5 (PMC8249521; doi:10.1038/s41598-021-93128-5)
Supplement: Supplementary file 1 — Supplementary Information 1. [file 41598_2021_93128_MOESM1_ESM.pdf]

Introducing the novel Cytoscape app TimeNexus to analyze time-series data using temporal MultiLayer Networks (tMLNs)

Michaël Pierrelée [1], Ana Reynders [2], Fabrice Lopez [3], Aziz Moqrich [2], Laurent Tichit [4] and Bianca H. Habermann [1]

Supplementary Data:

Supplementary methods

Notes to the selection of query nodes for the yeast cell cycle data and the mouse pain assay

We defined the query nodes by selecting the differentially expressed genes. Genes were called differentially expressed if they were deregulated between a time-point and a reference. The reference was the average gene expression across time for the yeast dataset and a control time-point for the mouse dataset. Comparing to a control time-point is relevant for perturbation experiments, where we measure the effects of a modified factor. When monitoring periodic processes (e.g. cell-cycle) or continuous processes (e.g. morphogenesis), choosing a particular time-point would be arbitrary, so deregulation can be defined by comparing either an expression level to an average expression, two consecutive time-points or expression levels using other statistical models. These models must be able to select the interesting genes at a given time-point as query nodes to guarantee that the extraction will prioritize them using TimeNexus together with either PathLinker or ANAT. Thereby, paying attention to the sample rate of time-series experiments is equally critical, because a given process cannot be studied with the same model when the sampling rates do not have the same order (e.g. minutes vs. hours vs. days).

Supplementary Tables:

Supplementary Table S1: Exemplary node table

Node	Weight_1	Weight_2	Weight_3	Query_1	Query_2	Query_3
YKL022C	0.039733842	0.031992741	0.028613951	FALSE	FALSE	FALSE
YGL116W	0.309084735	0.39682512	0.65252339	TRUE	TRUE	TRUE
YLR103C	0.395338334	0.293985868	0.274277839	TRUE	TRUE	TRUE
YMR001C	0.524117777	0.332445277	0.178371881	TRUE	TRUE	FALSE
YPR119W	0.548604495	0.399577103	0.202970354	TRUE	TRUE	FALSE
YPR120C	0.317746605	0.218357517	0.140080492	TRUE	FALSE	FALSE
YGR109C	0.561372231	0.343806274	0.222554583	TRUE	TRUE	FALSE
YMR199W	0.281998475	0.210768255	0.21303971	TRUE	FALSE	FALSE
YPL256C	0.412229379	0.295000464	0.288928797	TRUE	TRUE	TRUE

Supplementary Table S2: Exemplary intra-layer edge table

source	target	Weight	edge type
YKL022C	YLR102C	0.380438563	PPI
YKL022C	YLR127C	0.382194976	PPI
YKL022C	YMR001C	0.250513473	PPI
YGL116W	YML027W	0.25	PDI
YGR092W	YML027W	0.25	PDI
YLR103C	YLR274W	0.379473319	PPI
YLR103C	YMR043W	0.229153333	PPI
YLR103C	YPL153C	0.320794327	PPI
YLR103C	YPR019W	0.382440191	PPI
YLR127C	YMR001C	0.267785362	PPI
YLR127C	YNL172W	0.370272197	PPI

Supplementary Table S3: Exemplary inter-layer edge table

source	target	Weight_1>2	Weight_2>3
YKL022C	YKL022C	0.066926195	0.057143418
YGL116W	YGL116W	0.413802554	0.512040048
YLR103C	YLR103C	0.408047313	0.362352138
YMR001C	YMR001C	0.461370301	0.338106537
YPR119W	YPR119W	0.486700829	0.375993519
YPR120C	YPR120C	0.349002463	0.263860409
YGR109C	YGR109C	0.4751148	0.361577509
YMR199W	YMR199W	0.330102969	0.297658094
YPL256C	YPL256C	0.414255788	0.368658674

Supplementary Table S4: PathLinker optimization tests for different K (# of paths)

# of paths PathLinker	Subnetwork size	Recall	Precision	F1-score
**K50**	3.5	19.2	12.5	15.2
**K100**	5.6	29.2	11.7	16.7
**K150**	6.9	33.1	10.8	16.3
**K200**	7.4	36.9	11.2	17.1
**K250**	7.6	37.7	11.2	17.2
**K500**	8.6	40.8	10.6	16.9
**K750**	**9.6**	**45.4**	**10.6**	**17.2**
**K1000**	10.4	46.2	10	16.4
**K2000**	12.2	53.9	9.9	16.7

Supplementary Table S5: Excel sheet with data for Yeast cell cycle interactome (original node tables and intra-layer edge tables for early and late cell cycle phases, as well as enrichment results for early and late tMLNs

Supplementary Table S6: Excel sheet with node table, intra-layer -, and inter-layer edge table for entire yeast interactome with 16 cell cycle layers

Supplementary Table S7: relation of query nodes to KEGG cell cycle genes in queries of the original KEGG cell cycle tMLN

Query node-layers	# query nodes	# KEGG genes in query
**Layer 1**	243	26
**Layer 2**	148	18
**Layer 3**	242	20
**Layer 4**	193	14
**Layer 5**	132	11
**Layer 6**	135	13
**Layer 7**	172	23
**Layer 8**	249	29
**Layer 9**	264	32
**Layer 10**	281	34
**Layer 11**	178	10
**Layer 12**	98	4
**Layer 13**	100	6
**Layer 14**	87	5
**Layer 15**	62	2
**Layer 16**	69	2

Supplementary Table S8: PathLinker and AnatApp/ANAT Layer-by-Layer results

TN+extracting app	Subnetwork size (%)	Recall (%)	Precision (%)	F1-score (%)	percentage of expected GOs	percentage of top expected GOs
TN+PL_1	3.1	20.8	15.2	17.5	50	87.76
TN+PL_2	3.5	25.4	16.2	19.7	47.44	84.48
TN+PL_3	4.0	23.8	13.2	17.2	43.64	59.32
TN+PL_4	3.7	20.8	12.6	15.7	42.06	75.47
TN+PL_5	3.3	20	13.7	16.3	48.81	83.33
TN+PL_6	3.5	25.4	16.1	19.7	54.03	88.68
TN+PL_7	3.1	20	14.7	16.9	58.33	84.44
TN+PL_8	3.8	23.1	13.8	17.2	56.84	82.98
TN+PL_9	4.1	27	14.7	19	50.42	83.05
TN+PL_10	4.5	31.5	15.8	21.1	45.49	78.26
TN+PL_11	3.3	23.1	15.6	18.6	54.23	86
TN+PL_12	2.6	13.8	11.8	12.7	48.95	55.56
TN+PL_13	2.8	14.6	11.7	13	45.11	65.22
TN+PL_14	2.4	14.6	14	14.3	45.36	63.04
TN+PL_15	1.9	10	12.9	10.9	44.03	60.61
TN+PL_16	1.8	8.5	10.8	9.5	43.97	60
TN+ANAT_1	2.0	12.3	13.7	13	61.62	92
TN+ANAT_2	2.6	13.8	11.8	12.8	51.70	81.08
TN+ANAT_3	4.7	17.7	8.5	11.4	35.98	48.48
TN+ANAT_4	3.6	11.5	7.2	8.9	29.45	46.34
TN+ANAT_5	2.5	9.2	8.2	8.7	46.08	60
TN+ANAT_6	2.7	10.8	9.1	9.9	49.32	77.78
TN+ANAT_7	3.3	17.7	12.2	14.4	58.05	86.05
TN+ANAT_8	4.7	23.8	11.3	15.3	47.66	74.58
TN+ANAT_9	5	25.4	11.5	15.8	44.44	80
TN+ANAT_10	5.2	26.2	11.3	15.8	40.80	81.33
TN+ANAT_11	3.5	13.1	8.3	10.1	38.98	54.55
TN+ANAT_12	1.8	3.1	3.8	3.4	27.42	46.67
TN+ANAT_13	2.0	5.4	5.9	5.6	31.11	45.45
TN+ANAT_14	1.9	5.4	6.4	6.9	42.65	35.29
TN+ANAT_15	1.2	1.5	2.8	2	27.08	33.33
TN+ANAT_16	1.4	2.3	3.8	2.9	32.65	33.33

Supplementary Table S9: Excel table with original data on mechanical sensitivity assay, DEGs from mouse pain assay, node and intra-layer edge tables, plus GO enrichment of individual layers, we well as the entire extracted subnetwork

Supplementary Figures
Supplementary Figure S1

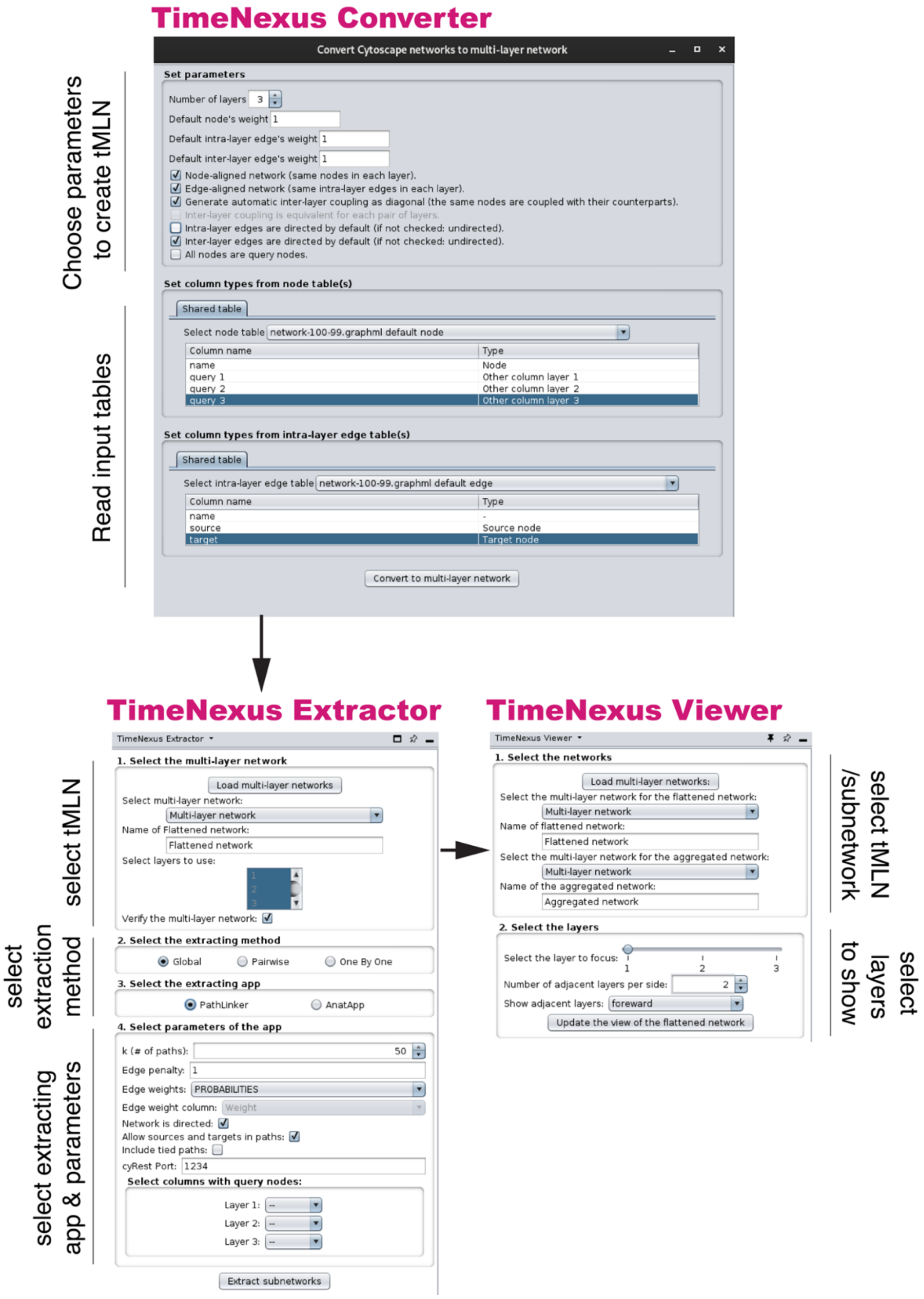

Figure S1 legend: The TimeNexus Converter, Extractor and Viewer interfaces. For creating a tMLN, first the table attributes have to be assigned to create the structure of the multilayer network using the **TimeNexus Converter**. In the **TimeNexus Extractor**, first a tMLN, second the extraction method and third the extracting app have to be chosen. The columns need to be individually assigned prior to extraction. The **TimeNexus Viewer** is needed to display a tMLN. Again, a tMLN has to be loaded and the layers to show have to be chosen.

Supplementary Figure S2

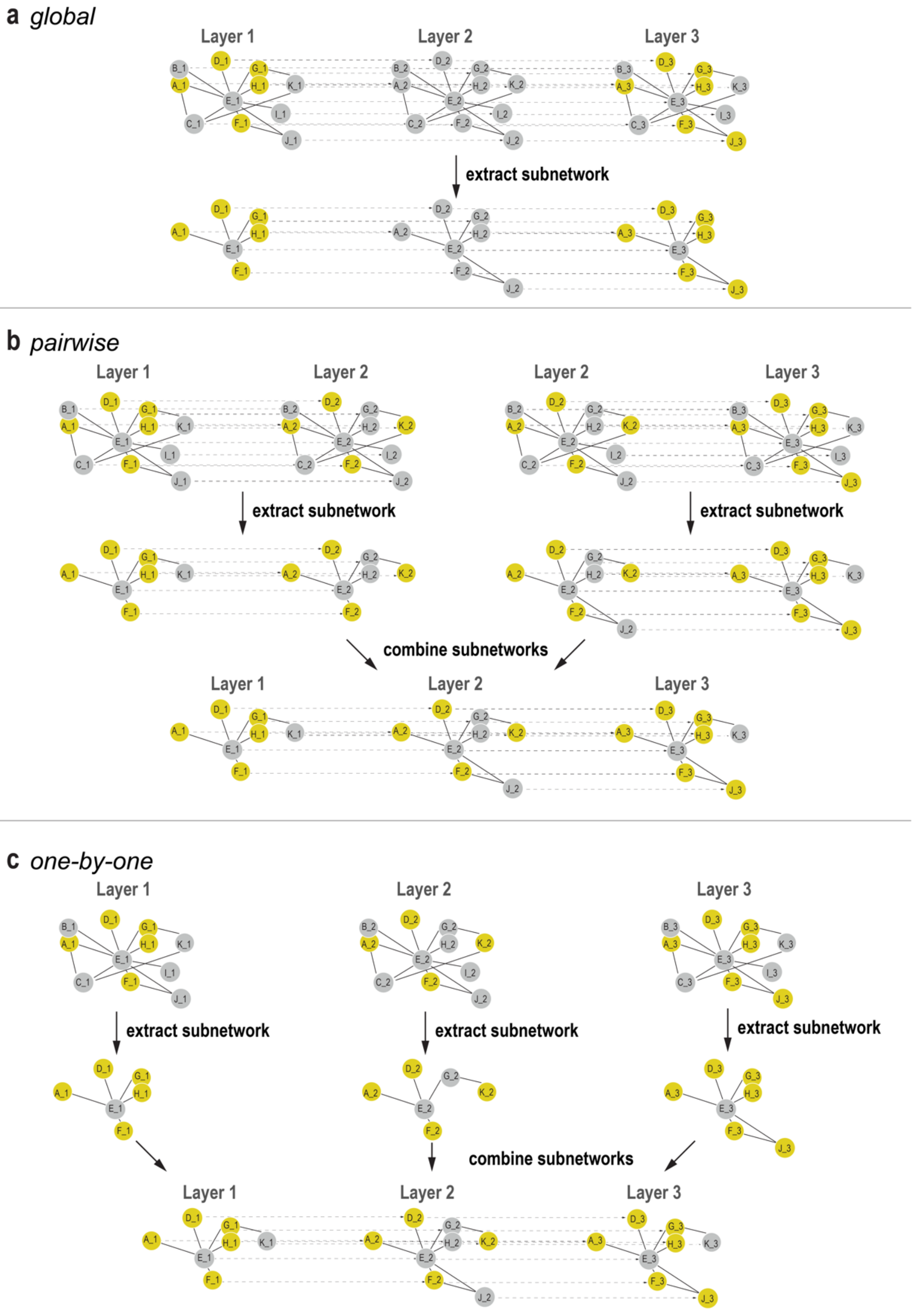

Figure S2 legend: Different extraction methods used in TimeNexus. (a) the *global* method extracts subnetworks over the entire flattened network-like structure, considering only the query nodes in the first and last layer. **(b)** in the *pairwise* method, two neighboring layers are collapsed for subnetwork extraction, whereby each layer is once layer N and once layer N+1. The subnetworks are combined to a final temporal multilayer subnetwork. **(c)** in the *one-by-one* method, one subnetwork is extracted per layer and all subnetworks are then combined to the final temporal multilayer subnetwork.
